# Obesity, type 2 diabetes, and testosterone in ageing men

**DOI:** 10.1007/s11154-022-09746-5

**Published:** 2022-07-14

**Authors:** Gary Wittert, Mathis Grossmann

**Affiliations:** 1 University of Adelaide, Adelaide, Australia; 2 Freemasons Centre for Male Health and Wellbeing, South Australian Health and Medical Research Institute, University of Adelaide, Adelaide, Australia; 3 Department of Medicine (Austin Health), The University of Melbourne, Heidelberg, Victoria4 Germany; 4 Department of Endocrinology, Austin Health, Heidelberg, VIC Germany; 5 South Australian Health and Medical Research Institute North Terrace Adelaide, 5000 SA Adelaide, Australia

**Keywords:** Testosterone, Ageing, Obesity, Type 2 diabetes, Men

## Abstract

In the absence of obesity, adverse lifestyle behaviours, and use of medication such as opioids serum testosterone concentrations decrease by only a minimal amount at least until very advanced age in most men. Obesity is heterogeneous in its phenotype, and it is the accumulation of excess adipose tissue viscerally associated with insulin resistance, dyslipidaemia, inflammation, hypothalamic leptin resistance and gliosis that underpins the *functional hypogonadism* of obesity. Both central (hypothalamic) and peripheral mechanisms are involved resulting in a low serum total testosterone concentration, while LH and FSH are typically in the normal range. Peripherally a decrease in serum sex hormone binding globulin (SHBG) concentration only partially explains the decrease in testosterone and there is increasing evidence for direct effects in the testis. Men with obesity associated functional hypogonadism and serum testosterone concentrations below 16 nmol/L are at increased risk of incident type 2 diabetes (T2D); high testosterone concentrations are protective. The magnitude of weight loss is linearly associated with an increase in serum testosterone concentration and with the likelihood of preventing T2D or reverting newly diagnosed disease; treatment with testosterone for 2 years increases the probability of a positive outcome from a lifestyle intervention alone by approximately 40%. Whether the additional favourable benefits of testosterone treatment on muscle mass and strength and bone density and quality in the long-term remains to be determined.

## Ageing and the hypothalamo-pituitary testicular (HPT) axis

Decreases in serum testosterone concentration are not an inevitable consequence of the ageing process in men [1] [2] [3]. The apparent effect of ageing on serum testosterone in Western populations is predominantly due to increasing visceral obesity, medication use and unhealthy lifestyle behaviours [4] [5]. Pathological disorders of the HPT axis such as Klinefelter syndrome which may not be recognised until later in life, affect at the most around 2–3% of men over the age of 40 years [4] [6].

## Obesity and obesity related chronic disorders and the HPT axis

In a metanalysis of 68 studies and almost 20,000 men with obesity, 42.8% had a serum total testosterone < 10.4 nmol/L (< 300ng/dl) [7]. Based on a mendelian randomisation analysis an increase of BMI from 25 to 30kgm^2^ is causally associated with a decrease in serum testosterone concentration of 13–15%. The reverse was not the case, and the effect was only partially dependent on an obesity related decrease in SHBG [8].

Dysfunction of the HPT axis that occurs in people with obesity and associated disorders, lifestyle behaviours. or medication use, is reversible with management directed to the causative factor(s). There is no inherent pathology of the HPT axis, and accordingly they are best thought of as “*functional disorders*” and accordingly referred to by the term *functional hypogonadism.*

### Obesity

Energy intake in excess to metabolic requirements is stored as lipid predominantly in adipose tissue. In obesity, excess adipose tissue is associated with actual or potential risks to health. Although body mass index (BMI) is commonly used as a measure of obesity, it does not provide information as to the distribution of body fat and the measure is confounded by variability in skeletal muscle mass. On average, beyond the age of 60 years, decreases in skeletal muscle mass occur, and unless accumulation of lipid filled adipose tissue is marked, the BMI curve flattens and then decreases. By contrast there is an incremental accumulation of visceral adipose tissue, most easily assessed by the measure of waist circumference (WC) which has a tighter association with the cardiometabolic associations of obesity than does BMI [9].

If excess adipose tissue is located only in subcutaneous depots, then adverse metabolic consequences or reduction in serum testosterone concentration generally do not occur. Excess fat around the abdominal viscera, infiltrating skeletal muscle and stored in the liver is associated insulin resistance, inflammation, and dyslipidaemia. It is in these circumstances, and typically in the presence of a large waist circumference that low serum testosterone concentrations occur [10, 11]. The association of visceral obesity associated HPT axis dysregulation is dependent on insulin resistance, increased serum leptin and triglycerides, reduced high density lipoprotein (HDL), the presence of hypertension, and non-alcoholic fatty liver disease (NAFLD) [12]. Among components of the metabolic syndrome, increased triglyceride levels and elevated blood pressure were independently associated with testosterone concentrations [12].

### Obesity related chronic disorders

In people with diabetes, glycaemic control does not appear to affect serum testosterone concentrations independent of obesity [13]. Obstructive sleep apnoea (OSA) which is bidirectionally associated with obesity is not independently associated with a low serum testosterone [14], and there is no independent effect of CPAP to increase serum testosterone in men with OSA [15, 16]. Obesity has been causally linked to depression [17, 18]. Some subtypes of depression may decrease testosterone [19]. In one study where depression was subtyped using latent class analysis serum testosterone concentrations were found to be significantly lower in men with an atypical depressive subtype than those with a more melancholic symptom profile [20].

### Health-related behaviours

Independent of weight, increased cardiovascular fitness [21, 22] and high levels of physical activity [23] are positively associated with higher serum testosterone concentrations. These observations are consistent with the well-established benefits of physical activity to ameliorate insulin resistance, obesity associated metabolic abnormalities, and inflammation independent of a change in overall weight. Cessation of smoking is associated with a decrease in serum testosterone concentration [4]. Heavy alcohol consumption may be associated with obesity and has testosterone-suppressive effects independent of body weight [24].

### Medications

Patients with obesity may be taking medications some of which, for example opioids [25] and to a minor extent statins [26] lower serum testosterone concentrations.

### Macronutrient intake

Macronutrient intake has also been shown to affect total testosterone concentrations in men independent of obesity status. At least acutely, meals of different macronutrient composition have been shown to have variable effects on serum testosterone concentration: decreased after poly- or mono-unsaturated fatty acids, increased after egg albumin, and unchanged after carbohydrate [27]. The postprandial decrease in serum testosterone has been shown to be greater in younger men and those who are not obese [28]. Based on cross sectional analyses of 208,677 community men aged 40–60 from the UK Biobank higher poultry and fish intake were associated with higher testosterone concentrations [23].

### Mechanisms of obesity related HPT axis dysregulation

#### Central mechanisms

In men with obesity related functional hypogonadism luteinising hormone (LH) is usually in the lower part of the normal range and inappropriate for the testosterone concentration. This indicates a central mechanism in the pathophysiology of obesity associated HPT axis dysregulation.

##### Leptin

Leptin is a cytokine hormone secreted by adipocytes in proportion the amount of fat stored and accordingly is a hormonal marker of energy sufficiency. Serum leptin concentration increases with obesity, but obesity is associated with resistance to the effects of leptin. Leptin is required for normal pulsatile secretory function of Gonadotrophin releasing hormone (GnRH) neurons in the arcuate nucleus of the hypothalamus (ARC). The permissive effects of leptin on GnRH pulsatility are mediated via kisspeptin expressing neurons in the ARC [29]. In mice with diet induced obesity, serum testosterone and LH concentrations decrease, and the serum concentration of leptin increases, while the expression of leptin receptor, kisspeptin and the kisspeptin receptor GPR54, in hypothalamic neurons decreases [30].

##### Pro-inflammatory cytokines

A state of low-grade inflammation has been causally linked to obesity associated HPT axis dysregulation. A transient inflammatory response induced by low dose endotoxin results in a decline in serum testosterone concentration, without changes in LH or FSH in lean men [31]. Treatment with an inhibitor of the inflammatory cytokine Interleukin-1 for 4 weeks increased serum testosterone concentrations by overall 0.96nmol/L (27.7ng/dL) compared to placebo. The greatest mean increases, 2.14nmol/L (61.7 ng/dL) and 2.64nmol/L (76ng/dL) occurred in those with C-reactive protein > 2 mg/L and those with BMI > 40 kg/m^2^, respectively [32]. The relatively modest treatment effect observed with an Interleukin-1 inhibitor in that study implies that other pro-inflammatory cytokines may have a pathogenetic role in obesity associated HPT axis suppression. Indeed, in the aforementioned study [31], the endotoxin induced decline in serum testosterone was associated with a significant increase in plasma interleukin-6 and tumour necrosis factor-alpha (TNF-a) concentrations. Moreover, in an experimental study of healthy men, infusion of the pro-inflammatory interleukin-2, even at relatively low doses, reduced the LH feedforward drive on testosterone secretion and inhibit GnRH and/or LH secretion [33]. Whether a more comprehensive suppression of the obesity associated proinflammatory state would lead to a more substantial increase in serum testosterone compared to a more cytokine specific treatment, e.g., with an Interleukin-1 inhibitor [32] has not been studied. While such studies would be of interest from a mechanistic perspective, given that mechanisms other than pro-inflammatory cytokines are operative in obesity associated HPT axis suppression, even a more comprehensive pharmacological suppression of circulating pro-inflammatory cytokines is unlikely to reverse obesity associated HPT axis suppression. Moreover, such studies would unlikely be of clinical relevance, given that in obese men, weight loss in itself has been associated with decreases in circulating concentrations of pro-inflammatory cytokines [34, 35].

##### Hypothalamic gliosis

In rodents, high fat diet overfeeding rapidly, and prior to obesity being measurable, induces neuronal inflammation resulting in insulin and leptin resistance in ARC neurons and subsequently fibrous proliferation of glial cells (gliosis) [36]. An inverse association between gliosis within the medio basal hypothalamus (but not putamen and amygdala control areas), and serum testosterone concentration has been reported in in men [37].

##### Oestradiol

Circulating sex steroids inhibit LH and GnRH secretion by the pituitary and hypothalamus respectively.

Testosterone inhibits LH by aromatisation to estradiol but has a direct effect on Gonadotropin Releasing. Hormone (GnRH). The negative feedback effect of estradiol is predominantly via GnRH [50]. Historical studies in markedly obese but otherwise healthy men have reported increased serum concentrations of circulating oestradiol [38]. Such studies led to the prevailing view that, via negative central feedback, increased circulating estradiol concentrations play a pathogenetic role in obesity associated HPT axis suppression. However, this notion is no longer correct, at least for men without WHO class III and IV obesity. More recent studies using accurate mass spectrometry measurements have reported reduced circulating concentrations of circulating estradiol in obese men, paralleling reductions of its substrate testosterone [39, 40]. In line with this, among obese men, lower levels of adipose tissue aromatase expression have been reported in those with a low circulating testosterone (defined in this study as a circulating free testosterone of < 174 pmol/L (< 5ng/dl) compared to controls with normal circulating testosterone [41]. Moreover, experimental studies have reported that oestradiol, rather than testosterone, is primarily important in preventing adiposity and insulin resistance in healthy men [42]. Of note, studies reporting that inhibiting oestradiol production or action via administration of aromatase inhibitors [43] or selective estrogen receptor modulators (SERMs) [44] increase LH and serum testosterone cannot be used to invoke a pathogenetic role for oestradiol in obesity associated HPT axis suppression, as similar effects have been reported in lean men [45]. Instead the testosterone response to aromatase inhibitors or selective estrogen receptor modulators merely reinforces that the obesity associated central hypothalamic-pituitary suppression is functional, as serum testosterone concentrations in men with organic congenital or acquired hypothalamic-pituitary pathology (such as congenital hypogonadotropic hypogonadism (e.g., Kalmann syndrome) or destructive mass lesions (e.g., pituitary macroadenoma)) do not increase in response to aromatase inhibitor or SERM treatment.

#### Peripheral mechanisms

*Effects on the testes*.

In rodent studies, high fat diet induced obesity has been shown to inhibit the synthesis of testosterone in testicular Leydig cells and recent studies have begun to elucidate the mechanisms by which this may occur. Accumulation of oxidized low-density lipoprotein (oxLDL) in Leydig cells of rodents disrupts mitochondrial function and inhibits the function of proteins and enzymes required for testosterone synthesis [46]. This may explain the why, at least in rats, Orlistat which inhibits gut lipase and consequently the absorption of fat, upregulates the expression of mRNA and protein of steroidogenic hormones and attenuates the decline that would otherwise been seen with obesity induced by high fat diet feeding [47]. In men, an increase in serum concentrations of oxLDL is independently associated with reduced serum testosterone [48].

Adenosine, a product of purine nucleotide and homocysteine catabolism has been implicated in the regulation of tissue damage in response to stressors, such as inflammation and hypoxia. In both diet-induced and genetically obese mice, adenosine accumulates in the testes, and has been causally linked to abnormalities of steroidogenesis in Leydig cells resulting in decreased production of testosterone [49].

Another mechanism recently implicated in obesity induced Leydig cell dysfunction in rodents with diet induced obesity is increased iron retention, possibly from dysregulation of a cellular hepcidin-ferroportin axis, occurs resulting in mitochondrial endoplasmic reticulum stress [50]. Iron accumulation in the testis inhibits the degradation of advanced glycosylation end products (AGEs) which have been implicated in obesity associated Leydig cell dysfunction [51].

Studies in rodents have reported that high fat diet induced obesity, compared to normal diet controls, is associated with a reduction in the testicular expression of genes involved in testosterone biosynthesis [52]. Moreover, in in vitro rodent studies, leptin has been reported to decrease both basal as well as human chorionic gonadotrophin (hCG)-stimulated testosterone secretion from rodent Leydig cells and from adult rat testes via a testicular leptin receptor mediated pathway [53, 54]. Moreover, in a cross-sectional study of men with BMIs from normal to severe obesity, circulating leptin was inversely correlated with testicular response to hCG stimulation [55]. These findings suggest that at least in part, the obesity associated decrease in serum testosterone concentration is due to decreased testicular production of testosterone [52]. Of note with respect to leptin these findings also imply that in obesity, there is differential tissue sensitivity to leptin, with both central leptin resistance (*see* Sect. 2.1.1.1*above*) and testicular sensitivity to leptin contributing to HPT axis suppression.

*Adipose tissue*.

Subcutaneous adipose tissue from men with obesity and insulin resistant murine 3T3-L1 adipocytes have increased T concentration with reduced lipolytic T release after adrenergic stimulation and are partially “resistant” to testosterone -mediated lipolysis and inhibition of adipogenesis, suggesting a bidirectional relationship between obesity and obesity associated decreases in serum testosterone concentrations [56].

*Sex hormone binding globulin variability*.

For testosterone to be transported via the circulation without degradation by enzymes, it must be carried by a binding protein. Testosterone binds predominantly to sex hormone binding globulin (SHBG) and to a lesser extent albumin, with high and low affinity respectively. SHBG is synthesised in hepatocytes of the liver A variable serum SHBG concentration is a source of the marked heterogeneity of serum testosterone concentrations among men with obesity [7]. Studies of SHBG gene regulation show that it is tightly linked to the regulation of hepatic lipid metabolism. Hormones (e.g., thyroid, growth hormone and oestradiol) and transcription factors (e.g., HNF1α) that promote fatty acid oxidation and inhibit lipid anabolism increase SHBG gene expression [57]. When hepatic de novo lipogenesis (DNL) increases, the expression and secretion of SHBG decreases, which explains the inverse relationship between high monosaccharide intake and SHBG [58]. Proinflammatory cytokines (TNFα and IL1b) decrease SHBG, effects mediated via by down-regulating HNF1α. The effect of insulin on SHBG expression is mediated indirectly via effects on hepatic lipid metabolism rather than directly on the mechanisms regulating the SHBG gene promoter. In the presence of hepatic insulin resistance oxidative metabolism of fatty acids decrease and de novo lipogenesis increases [57]. In accordance with these data we have shown, in a longitudinally followed cohort of men (n = 1736), that increasing serum thyroxine concentrations were positively associated with serum SHBG concentration and higher abdominal fat mass and serum triglycerides were inversely associated with serum SHBG concentrations [59].

## Clinical implications

### Serum testosterone and type 2 diabetes risk

The low serum testosterone associated with obesity is an independent risk factor for incident T2D [60]. The risk increases with severity of obesity and number of components of the metabolic syndrome [61]. The degree to which this is dependent on obesity remains inconclusive; in one longitudinal cohort study of 195 older (mean age 76 years) men, non-obese by BMI, followed for 8 years a low serum testosterone concentration at baseline was not a predictor of incident T2D [62]. Conversely in an earlier study, low baseline serum testosterone was predictive of developing incident metabolic syndrome over time, particularly in non-overweight, middle-aged men, with a BMI of < 25 kg/m^2^ [63]. Moreover, the predictive role of SHBG varies among observational studies with some [63, 64] but not all [60] studies reporting that low SHBG is predictive of the development of the metabolic syndrome and/ or incident T2D.

There is now accumulating evidence that explains the relationship between obesity, metabolic syndrome, testosterone, and risk of T2D in terms of high serum testosterone concentrations being protective. For example, in a metanalysis of 7 prospective studies (1966 to June 2005), the relationship between the serum testosterone concentration and T2D risk was linear up to a testosterone concentration of approximately 15.6 nmol/L (449.6ng/dL). Testosterone concentrations above this, up to 21 nmol/L (605.2 ng/dL), reduced the risk of T2D 42% [65]. Subsequently published prospective cohort data have shown that risk of T2D increased below a total testosterone of 16 nmol/L (461.4 ng/dL) and decreased at concentrations above that [66]. A more recent metanalysis of 10 prospective case control or nested cohort studies published between 1996 and 2016 showed a 38% reduction in T2D risk in men with higher serum testosterone [67]. Direct evidence is provided by the recently published T4DM (testosterone for the prevention of type 2 diabetes) study (discussed in detail below) which shows that the effect of treatment with testosterone to prevent T2D is independent of the baseline serum testosterone concentration [68].

### Effect of weight loss on obesity associated functional hypogonadism

In men with low serum testosterone attributable to obesity there is a linearly inverse association between decrease in weight and increase in serum testosterone (and SHBG) concentration [69].

*Diet induced weight loss*.

Modest diet induced weight loss (< 10%) prevents progression of prediabetes to T2D [70] and increases serum testosterone concentrations[71]. Greater magnitudes of weight loss induced by marked caloric restriction, for example using very low energy diets, may induce a remission of type 2 diabetes [72]. In contrast to an earlier study [73], a recently published meta-analysis has shown that the effects of weight loss induced by a hypocaloric diet, on testosterone and SHBG are not augmented by increased physical activity [74]. The nutrient composition of the diet may affect serum testosterone. A recently published metanalysis showed a significant decrease in serum testosterone concentration in response to high protein low carbohydrate diets [75]. However, there is marked heterogeneity among high protein diets with variation in the types of protein, carbohydrate, and fat, as well as the presence of fibre. In men who are overweight or obese, weight loss achieved with high protein, low carbohydrate diets and higher carbohydrate standard protein diets that have equivalent fibre and nutritional quality, improved testosterone, and SHBG to a similar extent [76]. Overall, the increase in serum testosterone achievable with diet alone is relatively modest, but not insubstantial; in a meta-analysis, low-calorie diet leading to a 9.8% reduction in body weight was associated with a 2.87 mmol/L (82.7 ng/dL) increase in serum testosterone [77]. However given that in most older men with obesity and/or T2D associated hypogonadism, serum testosterone concentrations are typically modestly reduced and fluctuate around the lower limit of the reference range [78, 79], even modest weight loss, although this may be difficult to sustain, can be associated with reactivation of the HPT axis [5] and in some men, biochemical normalisation of their androgen status.

*Bariatric surgery*.

Bariatric surgery produces substantial and durable weight loss with commensurate increases in serum testosterone SHBG, LH and FSH concentrations [80], and potentially sustained remission of T2D [81]. The effects of bariatric surgery on serum testosterone are substantial. In aforementioned metanalysis [77] bariatric surgery leading to a 32% reduction in body weight was associated with a 8.73 nmol/L (251.6 ng/dl) increase in serum testosterone. Of note however whether increases in serum testosterone associated with weight loss are causal mediators of the improvement in androgen deficiency-like features observed in some obese men following weight loss remains unproven; in one study that longitudinally followed obese men after bariatric surgery, the degree of weight loss, but the not the increase in serum testosterone was associated with improvements in sexual function [82].

*Pharmacotherapy*.

In men with T2D weight loss induced by the Glucagon-1-receptor agonists (GLP-1 RA) Exenatide was associated with an increase in serum testosterone only in men with lower serum testosterone concentrations at the outset [83]. In a 16-week clinical trial, treatment with the long acting GLP-1 RA Liraglutide, decreased weight, improved markers of the metabolic syndrome and increased serum testosterone concentrations whereas treatment with testosterone was without benefit [84]. Interestingly, an exploratory analysis of ‘The Researching Cardiovascular Events with a Weekly Incretin in Diabetes’ (REWIND) trial, a double-blind, placebo-controlled randomised trial of the effect of dulaglutide on cardiovascular outcomes, reported that, compared to placebo, dulaglutide may reduce the incidence of moderate or severe erectile dysfunction in men with T2D [85]. It is unclear whether these sexual benefits were a direct effect of the GLP-1 RA. Serum testosterone concentrations were not reported in this study. Weight loss has been shown to improved erectile function [86]. Of note, in contrast to testosterone treatment (*see* Sect. 3.3*below*), GLP-1RA have proven cardiorenal benefits in men with T2D.

### Is there a role for treatment with testosterone?

The T4DM study, a randomised double-blind placebo-controlled study, aimed to determine whether 2 years treatment with testosterone prevented T2D in men with obesity-associated functional hypogonadism. The study enrolled 1007 men aged between 50 and 74 years, with waist circumference over 94 cm, either impaired glucose tolerance or newly diagnosed T2D (20% of the total enrolled) established by an oral glucose tolerance test (OGTT) and a morning fasting serum testosterone of less than or equal to 14 nmol/L (403.5 ng/dL) as established by a platform chemiluminescent assay. All participants with serum testosterone < 8 nmol/L (230.5ng/dL) were assessed by an endocrinologist to exclude the presence of pathological hypogonadism, which if present was treated and excluded them from participation. Enrolled participants were randomised to 3 monthly intramuscular injections of 1000 mg of testosterone undecanoate or placebo on a one-to-one basis. All participants were enrolled in a WW (formerly Weight Watchers) program. At baseline and prior to each subsequent testosterone injection blood was drawn for the subsequent analysis of serum testosterone by LCMS. The primary endpoint was the proportion of participants with T2D at two years, assessed by oral glucose tolerance test (OGTT), representing either those who progressed from impaired glucose tolerance to T2D or those with newly diagnosed T2D who failed to regress [87]. OGTT-based measures (2-h glucose ≥ 11.1 mmol/L (199.8 mg/dL) and the mean change in the 2-h glucose on OGTT at 2 years compared with baseline) were chosen as the two co-primary outcomes as OGTT has better sensitivity and specificity for diagnosing T2D compared with fasting glucose and HbA1c, and represented a gold standard test as used in the Diabetes Prevention Program (DPP) [70]. It was also anticipated that testosterone would have an independent effect on HbA1c (*see below*).

Although there was an overall correlation between testosterone measurements obtained on baseline samples by LCMS and the screening chemiluminescent assay (CLIA), there was considerable variation among individuals. The spread of values on LCMS was 4 to 30nmol/L (115–864 ng/dL), and approximately 43% of participants had a baseline testosterone over 14nmol/L (493ng/dL) as measured by LCMS (unpublished data).

In testosterone as compared to placebo treated men there was a 40% reduction in the prevalence of T2D at two years, and a significant decrease in the 2 h glucose on the OGTT, improvement in glucose tolerance, and lower fasting glucose concentration. Consistent with other studies of testosterone treatment [88, 89], there was no effect on HbA1c. While the reasons for this finding are not fully understood, the utility of HbA1c as a marker of the glycaemic effects of testosterone treatment may be confounded by the erythropoietic actions of testosterone. While the effects of testosterone on red cell survival are not known, testosterone does increase red blood cell counts, and an increase in red cell survival could possibly explain the discrepancy between the OGTT and HbA1c findings in T4DM, although further studies are needed. In T4DM, the outcome was not dependent on either the screening or baseline testosterone concentrations. There were no differences in compliance with a lifestyle program between treatment groups and 70% of the men in each group achieved sufficient physical activity, which argues against an improvement in motivation with testosterone treatment. There was no significant difference in quality-of-life outcomes between the groups.

A notable outcome was that glucose tolerance at two years normalised in 43% of the men in the placebo group highlighting the efficacy of even a modest lifestyle intervention.

Testosterone treatment was also associated with beneficial changes in body composition, (decreased total and visceral fat mass, and increased skeletal muscle mass), muscle strength, and sexual function. In the placebo group fat mass, lean body mass and muscle strength all decreased [68]. Testosterone also improved bone density as assessed by DEXA. In a sub-group of participants (n = 177) assessed by high resolution-peripheral quantitative computed tomography (HR-pQCT) the predominant effect was an increase in cortical area and thickness with only minimal effects on trabecular bone accompanied by a reduction in bone remodelling markers [90].

There were no overall differences in serious adverse events between the groups. Cardiac and cerebrovascular safety were particularly reassuring. A similar number of placebo and testosterone treated men were diagnosed with prostate cancer over the 2 years of the study; there were fewer high-grade cancers in the testosterone treated men. There were 106 (21%) testosterone treated men who developed a haematocrit greater than 0.54%. However, only 25 of these men had treatment withdrawn prior to the final testosterone dose [68]. It should be noted that obstructive sleep apnoea (OSA) did not preclude enrolment in T4DM [87]. Approximately 25% of men over the age of 40 years have moderate to severe OSA and the proportion is higher with increasing age and obesity [91]. Although speculative, it seems likely that the relatively high frequency of increased haematocrit in this population of testosterone treated men is attributable to underlying untreated OSA.

In contrast to the benefits of testosterone treatment observed in the T4DM study, a recent 12-week trial showed that over weeks, exercise but not testosterone had a benefit on vascular function and health [92]. As discussed elsewhere in this issue, the long-term effects of testosterone treatment on cardiovascular health remain uncertain, due to the current lack of availability of dedicated cardiovascular outcome trials powered for clinical cardiovascular events.

### Mechanism of effect of testosterone on glucose metabolism

Glucose tolerance depends on the interplay between cellular mechanisms of glucose uptake and utilisation and insulin production by pancreatic beta cells.

There is some evidence to show that testosterone increases glucose stimulated insulin secretion from human cadaveric islets [93], a process dependent on the presence of 5 alpha reductase and possibly aromatase in pancreatic beta cells [94]. However, there are no data to show that acute or short-term administration of testosterone to hypogonadal men increases glucose dependent insulin production.

The potential effects of testosterone on insulin signalling and glucose utilisation have recently been reviewed [95]. In adipose tissue testosterone increases insulin receptor β subunit, insulin receptor substrate-1, protein kinase B and glucose transporter type 4 expression. In skeletal muscle testosterone increases adenosine 50-monophosphate-activated protein kinase expression and activity. Testosterone treatment also decreases markers of inflammation linked and free fatty acids linked to inhibition of insulin signalling [95].

In men with functional hypogonadism and type 2 diabetes, topical testosterone treatment for 24 weeks, but not 3 weeks, improved insulin sensitivity an effect associated with a decrease in adipose tissue mass. Free fatty acids decreased from 15 weeks, but any effect on insulin sensitivity could not be disentangled from the change in body composition [96]. Treatment of men with prediabetes [97] or type 2 diabetes [98] and functional hypogonadism with 3 monthly intramuscular testosterone undecanoate injections shows a cumulative time dependent effect to decrease waist circumference and other anthropometric obesity indices paralleled by progressive improvements in glucose tolerance.

Taken together these data suggest that the mechanism by which testosterone treatment improves glucose metabolism in men is dependent on favourable changes in body composition. In accordance with this, a preliminary mediation analysis of data from the T4DM study suggests that the beneficial effects of testosterone on glucose metabolism in was predominantly (but not entirely) mediated by decreased fat mass [68]. Ongoing analyses from this study may provide further clarification as to the mechanisms by which testosterone produces beneficial effects on glucose metabolism.

### Implications for practice

- Low serum testosterone concentrations with increasing age are predominantly the consequence of obesity associated chronic disease, opioid use, and modifiable health-related behaviours.
- The aim of clinical assessment must be to exclude the presence of pathological hypogonadism, identify and effectively manage co-morbid physical and psychological chronic conditions and provide education and support to optimise health-related behaviours. Such measures have been shown to treat or prevent chronic disease, increase serum testosterone concentration, and improve sexual function.
- Treatment with testosterone in combination with a lifestyle program in centrally obese men with impaired glucose tolerance or newly diagnosed T2D can decrease fat mass, improve glucose tolerance, and prevent or reverse recently diagnosed type 2 diabetes.
- The durability of benefit and longer-term safety of testosterone used as a pharmacological approach for T2D prevention in men with obesity-associated functional hypogonadism, and whether there are subgroups of men who will benefit, remain to be determined.
- It is not known whether the testosterone induced increase in bone and skeletal muscle mass and grip strength will reduce the risks of subsequent fracture or frailty.
